# DNA Hypermethylation of *CREB3L1* and *Bcl-2* Associated with the Mitochondrial-Mediated Apoptosis via PI3K/Akt Pathway in Human BEAS-2B Cells Exposure to Silica Nanoparticles

**DOI:** 10.1371/journal.pone.0158475

**Published:** 2016-06-30

**Authors:** Yang Zou, Qiuling Li, Lizhen Jiang, Caixia Guo, Yanbo Li, Yang Yu, Yang Li, Junchao Duan, Zhiwei Sun

**Affiliations:** 1 Department of Toxicology and Sanitary Chemistry, School of Public Health, Capital Medical University, Beijing 100069, P.R. China; 2 Beijing Key Laboratory of Environmental Toxicology, Capital Medical University, Beijing 100069, P.R. China; Institute of Biochemistry and Biotechnology, TAIWAN

## Abstract

The toxic effects of silica nanoparticles (SiNPs) are raising concerns due to its widely applications in biomedicine. However, current information about the epigenetic toxicity of SiNPs is insufficient. In this study, the epigenetic regulation of low-dose exposure to SiNPs was evaluated in human bronchial epithelial BEAS-2B cells over 30 passages. Cell viability was decreased in a dose- and passage-dependent manner. The apoptotic rate, the expression of caspase-9 and caspase-3, were significantly increased induced by SiNPs. HumanMethylation450 BeadChip analysis identified that the PI3K/Akt as the primary apoptosis-related pathway among the 25 significant altered processes. The differentially methylated sites of PI3K/Akt pathway involved 32 differential genes promoters, in which the *CREB3L1* and *Bcl-2* were significant hypermethylated. The methyltransferase inhibitor, 5-aza, further verified that the DNA hypermethylation status of *CREB3L1* and *Bcl-2* were associated with downregulation of their mRNA levels. In addition, mitochondrial-mediated apoptosis was triggered by SiNPs via the downregulation of PI3K/Akt/CREB/Bcl-2 signaling pathway. Our findings suggest that long-term low-dose exposure to SiNPs could lead to epigenetic alterations.

## Introduction

The nanotechnology industry has grown exponentially over the last decade in a diverse range of applications, including medicine (therapeutic, diagnostic and bio-imaging), food ingredients, cosmetics, and electronics [1,2]. More than 1600 consumer products containing nanomaterials are currently available in our daily life [3]. According to reports in the Project on Emerging Nanotechnologies, silica nanoparticles (SiNPs) are listed within the Top 3 nanomaterials-based consumer products [4]. With the growing number of applications for SiNPs, the potential burden on human and environmental exposure are increasing. Humans can be exposed to SiNPs via inhalation, dermal penetration ordigestion [5], thus, it is crucial to assess their potential adverse biological effects. In vitro and in vivo studies have revealed that SiNPs can cause cytotoxicity, genotoxicity, cardiovascular toxicity, pulmonary toxicity and hepatotoxicity [6–11]. Yet, there are only very few studies that investigate nanomaterials-induced epigenetic toxicity [12], and especially limited for SiNPs in particular.

Generally, epigenetic regulation of gene transcription occurs by three main mechanisms: DNA methylation, histone modification and miRNA expression [13]. DNA methylation, the most common epigenetic mechanism, leads to changes in gene expression without alteration of DNA sequences [14]. Aberrant (hyper- or hypo-) methylation is believed to be greatly influenced by environmental risk factors, resulting in physiological instability of cell division [15,16]. Hypermethylation of promoter regions (CpG islands) silences genes involved in DNA repair, cell cycle and apoptosis pathways; while hypomethylation of a CpG dinucleotide in the global DNA sequence activates gene expressions [17]. Numerous studies have explored the genotoxic potential of nanomaterials, yet, very few studies have assessed their potential for epigenetic regulation [12]. Choi and coworkers first reported that nanomaterials could induce significant epigenetic changes in 2008, by demonstrating that CdTe quantum dots (QDs) decreased DNA methylation of specific apoptotic and antioxidant genes in human MCF-7 breast cancer cells [18]. More recently, titanium dioxide nanoparticles were shown to increase the levels of DNA methylation in the PARP-1 promoter in A549 cells [19]. In contrast, no changes in DNA methylation were observed in Neuro-2A cells exposed to copper oxide nanoparticles [20]. The other types of epigenetic modifications in EK cells exposure to nanoparticles were also reported: Eom et al. found that the differential sensitivity of integrated mRNA and microRNA profiling in Jurkat T cells exposed to AgNPs and Ag ions [21]. Produced significant changes in microRNA expression were also found in different size of gold nanoparticles [22]. Apart from these isolated report, there is a scarcity of information on nanomaterials-induced epigenetic mechanisms, with the limited lack of consistency conclusions that can be drawn.

In the present study, epigenetic regulation of low-dose SiNPs exposure was first evaluated in human bronchial epithelial BEAS-2B cells over 30 passages. We adopted the HumanMethylation450 BeadChip to analyze genome-wide methylation profiles. The cytotoxicity, apoptosis, and activation of caspase-3 and caspase-9 were evaluated after BEAS-2B cells treated with SiNPs. Microarray data indicated the involvement of the PI3K/Akt/CREB/Bcl-2 signaling pathway which was further verified by qRT-PCR and western blot assays. In addition, the methyltransferase inhibitor5-aza-2’-deoxycytidine (5-aza), was performed to analyze the role of SiNPs on DNA methylation and mRNA levels of the apoptosis-related genes *CREB3L1* and *Bcl-2*.

## Materials and Methods

### SiNPs preparation and characterization

SiNPs were prepared and characterized as described in our previous studies [23]. Briefly, 2.5 mL of tetraethylorthosilicate (TEOS) (Sigma, USA) was added to premixed ethanol (50 mL) containing ammonia (2 mL) and water (1 mL). The reaction mixture was kept at 40°C for 12 h with continuous stirring (150 r/min). Particles were isolated by centrifugation (12,000 r/min, 15 min) and rinsed three times with deionized water. SiNPs were dispersed in deionized water (50 mL) by sonicator for 5 minutes (160 W, 20 kHz, Bioruptor UDC-200, Belgium) prior to experiments using in culture medium to minimize its aggregation.

### Cell culture experiment and exposure to SiNPs

Human Bronchial epithelial cells (BEAS-2B) lines were purchased from the Cell Resource Center, Shanghai Institutes for Biological Sciences (SIBS, China). Cells were cultured in Dulbecco’s Modified Eagle’s Medium (DMEM) (Hyclone, USA) supplemented with 10% fetal bovine serum (Gibco, USA), 100 U/mL penicillin and 100 mg/mL streptomycin, in a humidified environment (37°C, 5% CO_2_). For all experiments, the cells were seeded in culture plates at a density of 1×10^5^ cells/mL, allowed to attach for 24 h, and then treated with SiNPs (suspended in DMEM) for 24 h. Cells were collected after 1 passage, and the SiNP-treated process was repeated over 30 passages. Control treatment cells were provided with an equivalent volume of DMEM without SiNPs. Each group had three replicate wells.

### MTT assay

The cell viability was determined using the MTT assay. Briefly, 1×10^4^ BEAS-2B cells were seeded on a 96-well plate in a volume of 100 μL DMEM, and allowed to attach for 24 h at 37°C. Cells were treated with varying concentrations of SiNPs (3.125, 5, 6.25, 12.5, 25, 50 and 100 μg/mL) for 24 h at 37°C. MTT (10 μL) was added into each well at 5 mg/mL. Following 4 h incubation, 150 μL dimethylsulfoxide (DMSO) was added and mixed thoroughly for 5 min. Optical density at 492 nm measured with a microplate reader (ThemoMultiscan MK3, USA). The concentration of SiNPs (5 μg/mL) that exhibited greater than 95% cell viability was chosen for long-term low-dose SiNPs exposure. Cell viability was also measured at the 5th, 10th, 20th and 30th passages.

### Apoptosis assay

Apoptosis in BEAS-2B cells was assessed using an Annexin V-propidium iodide (PI) apoptosis detection kit (Jiancheng, China) according to the manufacturer’s instructions. Briefly, BEAS-2B cells were exposed to SiNPs for 30 passages, washed with PBS three times and trypsinized. After centrifugation at 1000 rpm, the cell pellet was washed with PBS once and incubated with 5 μL Annexin V-FITC for 15 min, followed by staining with 5 μL PI. Samples were diluted with 500 μL binding buffer and analyzed with a flow cytometer (Becton Dickinson, USA), counting at least 1×10^4^ cells for each sample.

### Methylation BeadChip array

DNA was isolated using a micro DNA isolation kit (Qiagen, Valencia, CA) according to the manufacturer’s instructions. DNA (500 ng) was treated with bisulfate using an EZ DNA Methylation Gold Kit (Zymo Research, Irvine, CA), according to the manufacturer’s instructions. The methylation of DNA was assayed on Infinium HumanMethylation450 BeadChips using the Illumina HD methylation assay kit from Shanghai Biotechnology Corporation.

Data analysis for the methylation BeadChip arrays was carried out by extracting image data using the Genome Studio methylation module. β-values (ranging from 0 to 1), reflecting significantly differentially methylated sites (i.e. ∆β≥0.14 and *p*≤0.05) were identified in different tissue types. Genes were grouped in functional categories based on the Gene Ontology database (GO: http://www.geneontology.org/), and functional pathways (KEGG and BIOCARTA) were analyzed using the online SAS analysis system (http://www.ebioservice.com/sas.html).

### Pyrosequencing analysis

DNA samples underwent bisulfite conversion using the EpiTect Plus Bisulfite Kit(Cat#59104, Qiagen, Germany) following treatment of BEAS-2B cells with the methyltransferase inhibitor 5-aza for 24 h. Pyrosequencing and PCR amplification of CREB3L1 and Bcl-2 were conducted using the PyroMark assay design software and PyroMark PCR kit, respectively. All processes were performed according to manufacturer’s instructions at Shanghai Biotechnology Corporation. Primers for the Pyrosequencing analysis are listed in S1 Table.

### Quantitative RT-PCR analysis

Total cellular RNA was extracted using TRI Reagent (Molecular Research Center) according to the manufacturer’s instructions. Reverse transcription (RT) of the purified total RNA was performed using the iScriptcDNA synthesis kit (Bio-Rad 1708891, USA). The expression level of GAPDH was used as an internal control. qRT-PCR was performed in a 7900 HT Sequence Detection System (ABI, USA) using the Power SYBR Green PCR Master Mix (ABI, USA).The relative gene expression was analyzed using the 2^−(ΔΔCt)^ method and normalized to the control. All experiments were performed in triplicate, three independent times. The primers used for qRT-PCR are listed in S2 Table.

### Western blot analysis

Equal amounts (40 μg) of lysate proteins were loaded on 12% SDS-polyacrylamide gels and electrophoretically transferred onto polyvinylidene fluoride (PVDF) membranes (Millipore, USA). After blocking with Tris-buffered saline (TBS) containing non-fat milk (5%) for 1 h, membranes were incubated with caspase-9, caspase-3, protein kinase B (Akt), p-Akt, cAMP responsive element-binding protein (CREB), or Bcl-2 (CST, USA) (1:1000, rabbit antibodies) at 4°C overnight. The PVDF membrane was rinsed with TBST and incubated with anti-rabbit IgG secondary antibody (CST, USA) for 1 h. Proteins bound to antibodies were measured by the chemiluminescence reagent, ECL (Pierce, USA). Densitometric analysis of the western blot results was assessed using Image Lab™ Software (Bio-Rad, USA).

### Statistical analysis

Statistical analysis was performed using SPSS 18.0 software (SPSS Inc., Chicago, IL, USA). Data were expressed as mean ± standard deviation of independent experiments. The least significant difference (LSD) test was used to compare the means of two samples, while one-way analysis of variance (ANOVA) was used for multiple comparisons of >2 groups. In all cases, *p*<0.05 was considered to be statistically significant.

## Results

### Characterization of SiNPs

Full characterization of SiNPs can be found in our previous study [21]. Briefly, the average diameter of SiNPs was approximately 58 nm. The hydrodynamic sizes and zeta potentials of SiNPs were detected in the distilled water and DMEM. The present results showed that SiNPs possessed favorable dispersibility and stability in the culture medium. And the purity of SiNPs was more than 99.9%.

### Cytotoxicity of SiNPs

**Fig 1:**
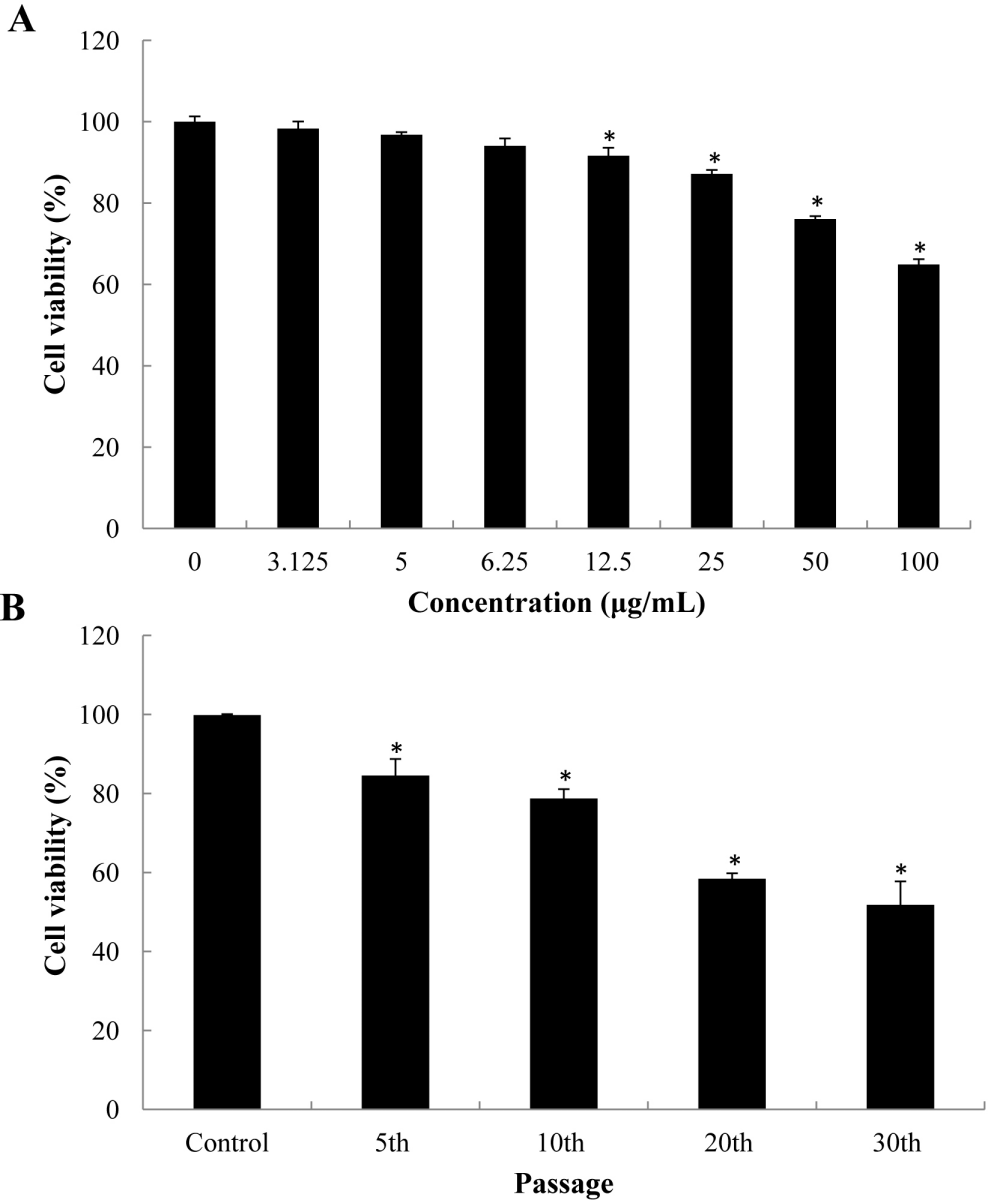
Cell viablity of BEAS-2B cells in the presence of SiNPs. (A) Cell viability of 3.125, 5, 6.25, 12.5, 25, 50, 100μg/mL SiNPs-treated groups for 24 h exposure. (B) Cell viability following 5 μg/mL SiNPs after the5th, 10th, 20th, 30th passage. Data are expressed as means ± S.D. *p<0.05 compared with control group.

After 24 h SiNPs exposure, the viability of BEAS-2B cells decreased gradually in a dose-dependent manner (Fig 1A). The cell viability of 12.5, 25, 50, 100 and 200 μg/mL SiNPs-treated groups were significant decreased compared to control. The concentration of SiNPs (5 μg/mL) that retained >95% cell viability was chose for long-term exposure dosage. The cell viability of SiNPs was decreased to 84.5%, 78.7%, 58.4%, 51.8% at the 5th, 10th, 20th, 30th passage, respectively (Fig 1B). Our data showed that SiNPs induced the cytotoxicity in a dose- and passage-dependent manner.

### Mitochondrial apoptosis induced by SiNPs

**Fig 2:**
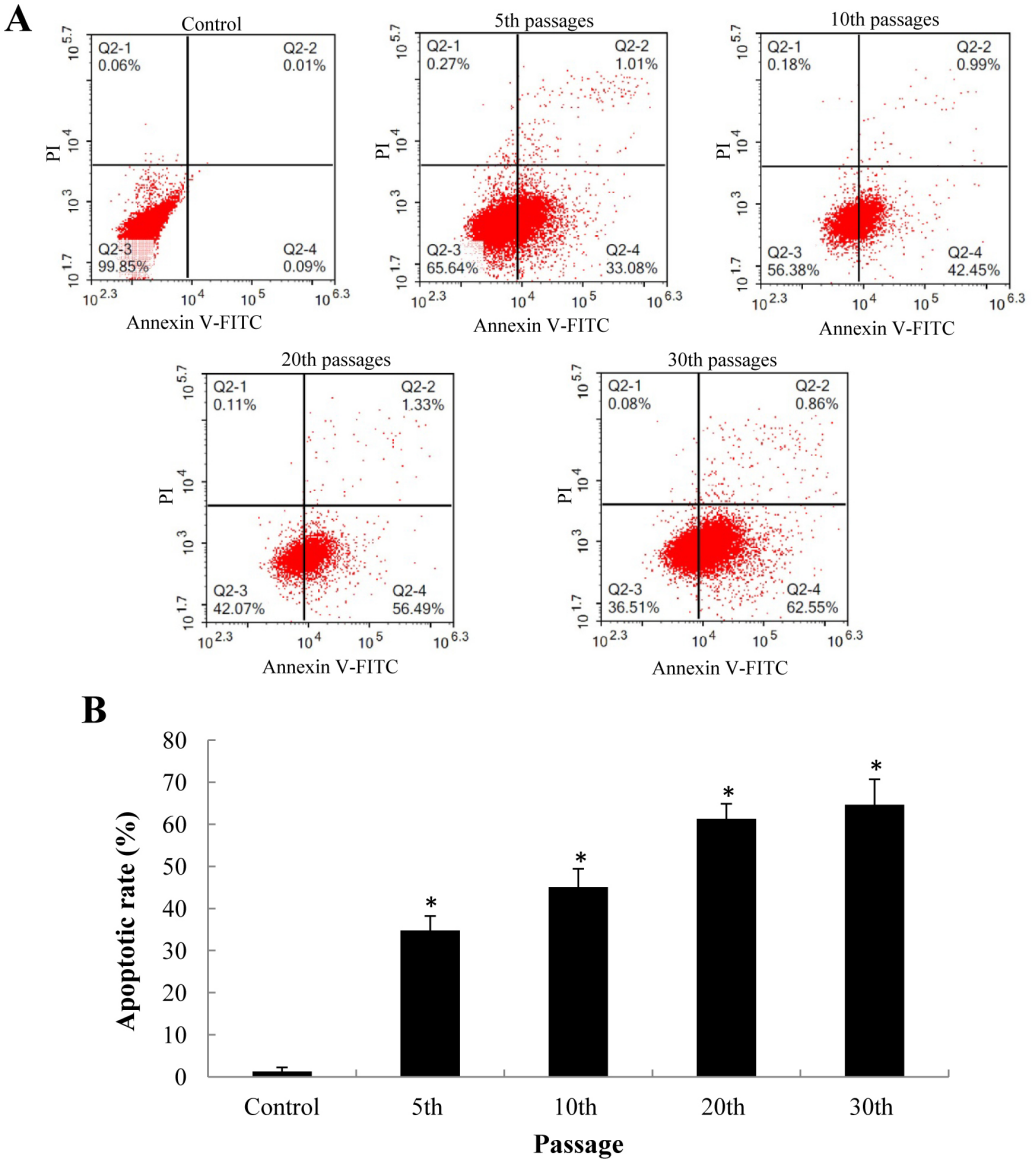
Apoptosis of BEAS-2B cells after exposure to SiNPs at the 5th, 10th, 20th, 30^th^ passage. (A) Apoptotic populations of cells double-stained with PI- and FITC-labled Annexin V using flow cytometry. (B) SiNPs increased the apoptosis rate in a passage-dependent manner. Data are expressed as means ± S.D. *p<0.05compared with control group.

**Fig 3:**
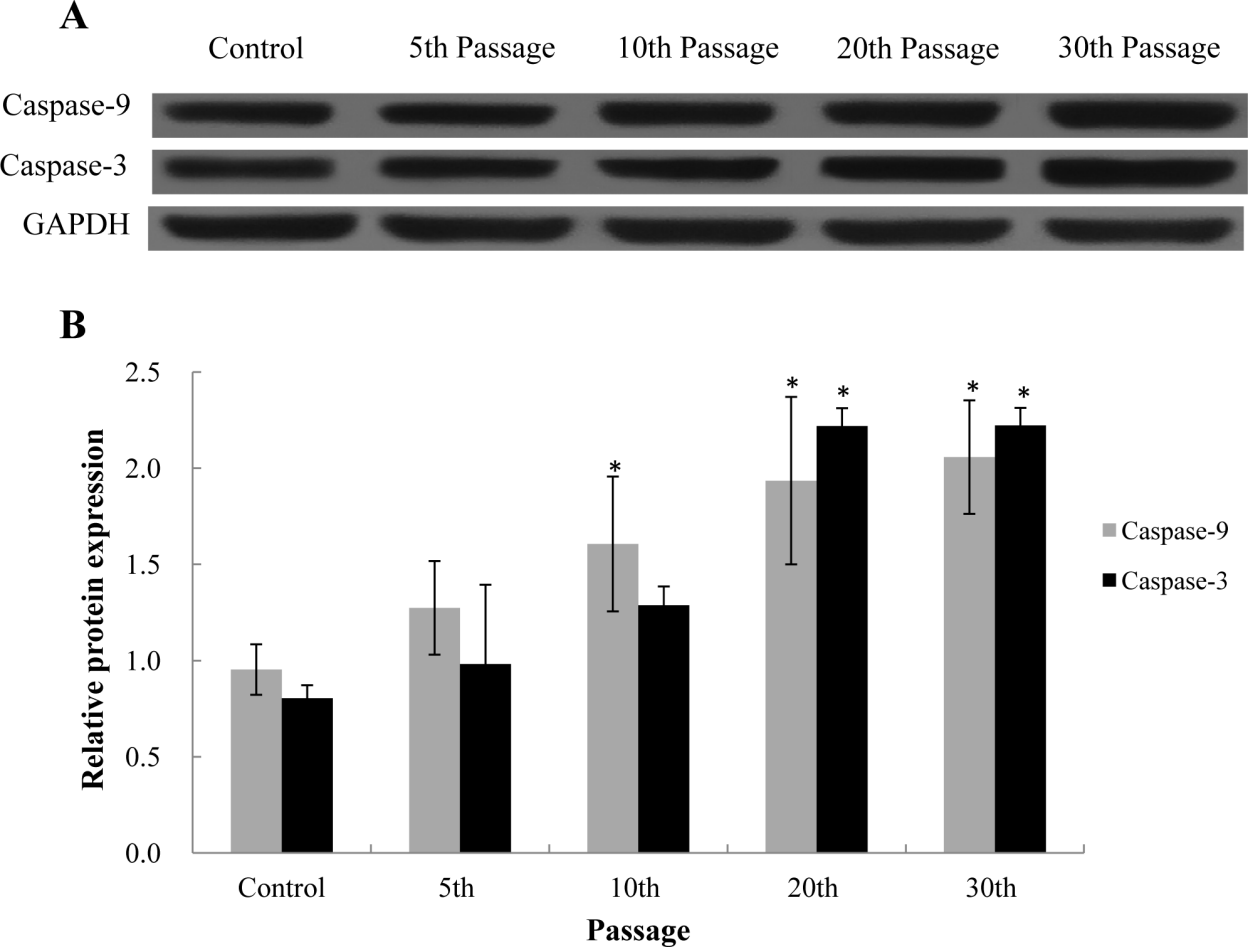
Effect of SiNPs on the expression of caspase-9 and caspase-3 in BEAS-2B cells. (A) Protein levels induced by SiNPs at the 5th, 10th, 20th, 30th passage. (B) Relative densitometric analysis. Data are expressed as means ± S.D. *p<0.05 compared with control group.

To further explore the nature of cell death induced by SiNPs, apoptosis was measured by flow cytometry. The apoptotic rate was significant higher in SiNP-treated groups compared to that of control at the 5th passage (Fig 2). The apoptotic rate induced by SiNPs at 5th, 10th, 20th, 30th passages were 34.8%, 45.1%, 61.3%, 64.6%, respectively. The activities of caspase-9 and caspase-3, were assessed by western blot assay (Fig 3). Relative densitometric analysis showed that the expression of Caspase-3 and Caspase-9 was significant increased in SiNPs-treated groups and the protein level of Caspase-3 and Caspase-9 was markedly upregulated. Our data demonstrated that the SiNPs could trigger the mitochondrial apoptosis in a passage-dependent manner in BEAS-2B cells.

### Identification of DNA methylation status of global genes induced by SiNPs

**Fig 4:**
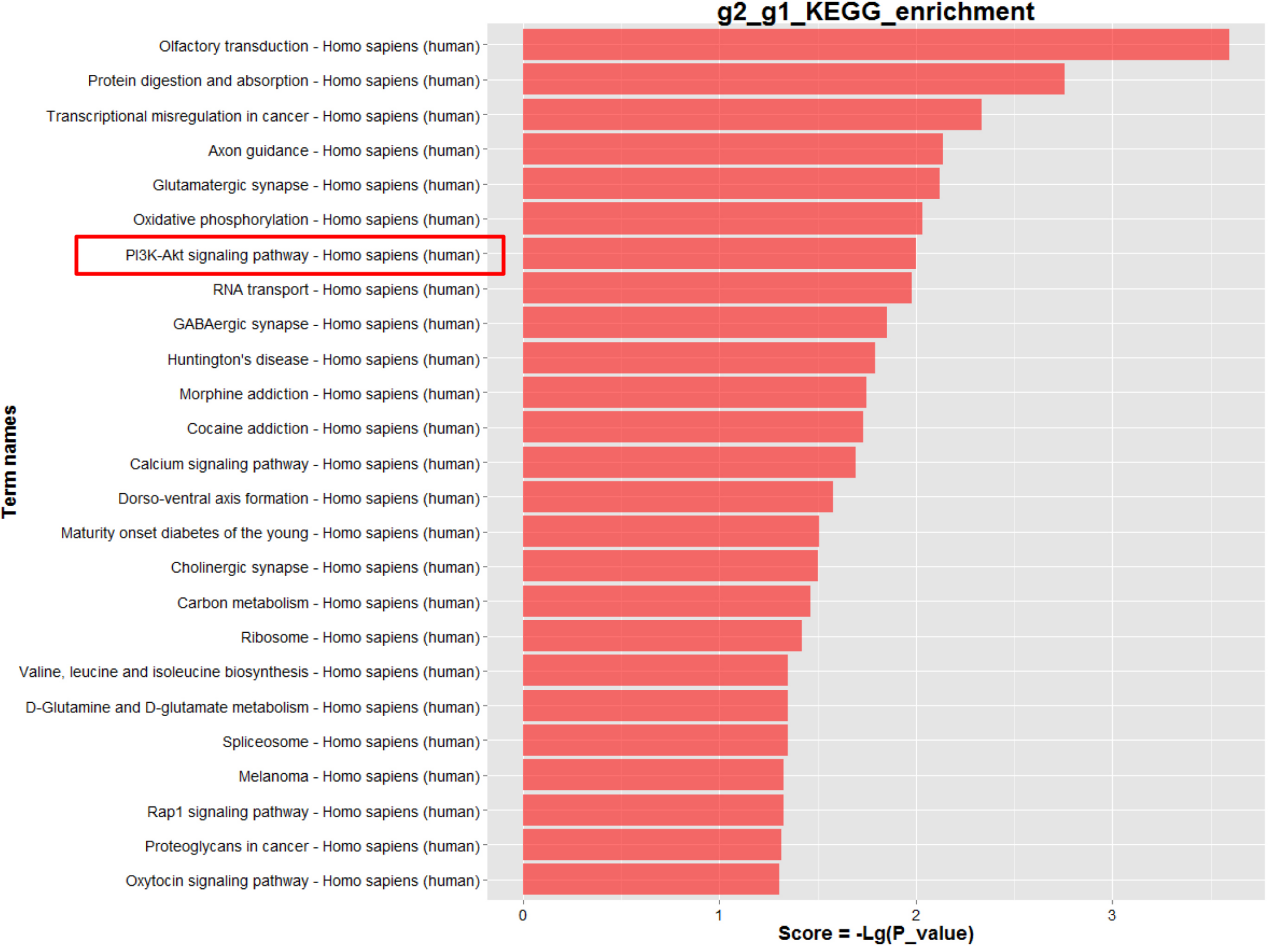
Analysis of gene DNA methylation status between the SiNPs-treated BEAS-2B cells (g2) and control cells (g1) using Infinium HumanMethylation450 BeadChip.

To investigate whether long-term SiNPs exposure resulted in the alteration of the DNA methylation status in BEAS-2B cells, we adopted theHumanMethylation450 BeadChip. Among the 45,000 CpG loci, there were 2,196 CpG loci that showed differential methylation levels (β-values) between the SiNPs-BEAS-2B and BEAS-2B (*p*<0.05). Of these 2,196 CpG loci, 223 were considered to be hypomethylated (Δβ<-0.14) and 1,973 were considered to be hypermethylated (Δβ > 0.14) (Fig 4). After enrichment analysis of KEGG (*p*<0.05), the methylated sites were associated with a total of 25 pathways (Fig 4). The PI3K/Akt pathway was identified as the apoptosis-related pathway among the 25 significantly altered pathways. Within the PI3K/Akt signaling pathway, the differentially methylated sites involved 32 different gene promoters, in which the *CREB3L1* and *Bcl-2* gene were significant hypermethylated.

### Hypermethylation of *CREB3L1* and *Bcl-2* promoters induced by SiNPs

**Fig 5:**
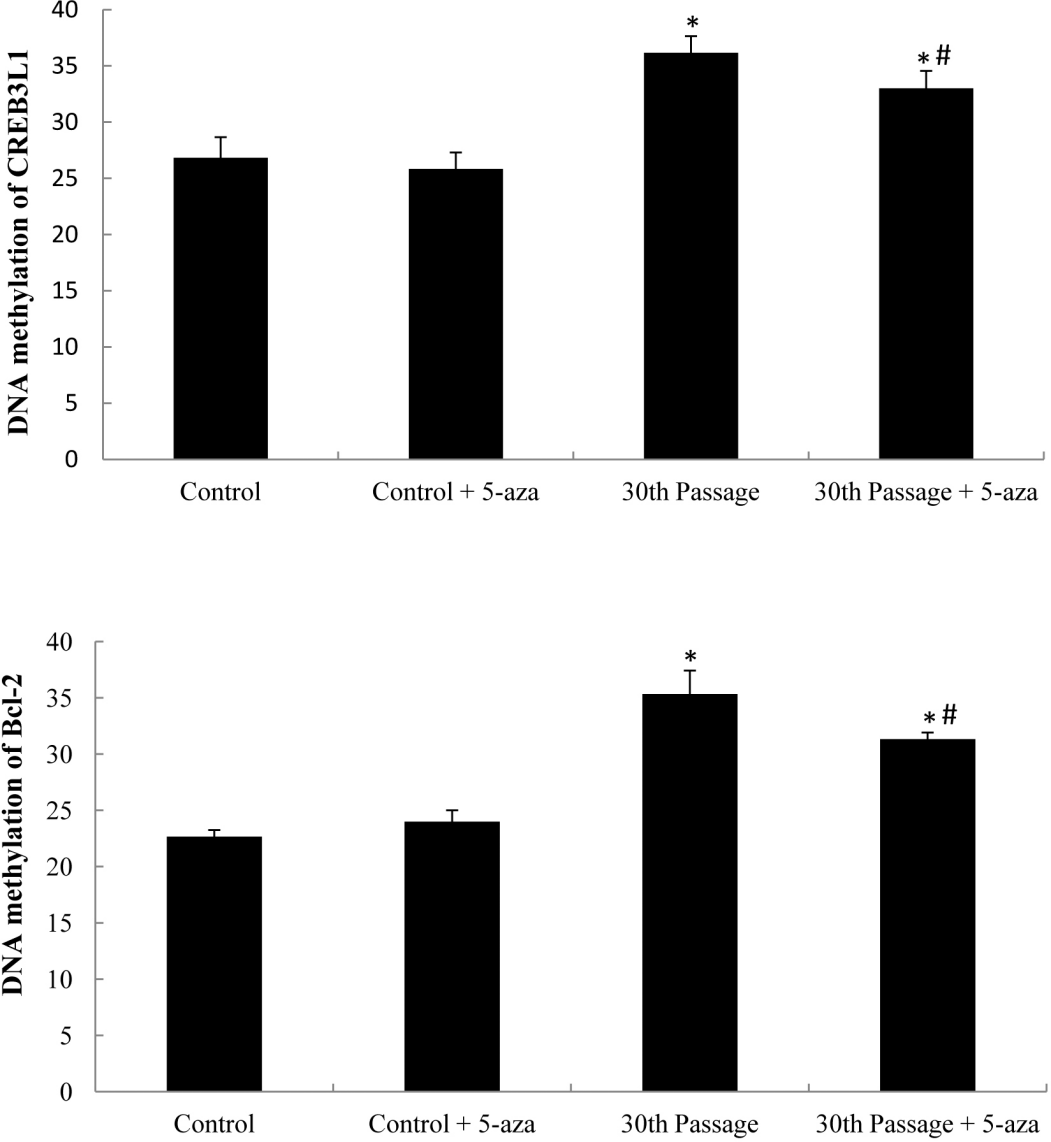
DNA methylation of *CREB3L1* and *Bcl-2* promoters in the 30th passage of BEAS-2B cells exposed to SiNPsin the presence of the methyltransferase inhibitor 5-aza. Data are expressed as means ± S.D. *p<0.05 compared with control group. ^#^p<0.05 compared with the 30th passage SiNPs-treated group.

We analyzed normal BEAS-2B cells and the 30th passage of SiNPs-exposed BEAS-2B cells by Illumina methylation array. Among all CpG bb sites that were hypermethylated, there was DNA methylation of CpG sites located in the N-shore of *CREB3L1* gene, and CpG sites located in the island of the *Bcl-2* gene (S3 Table). The methylation of *CREB3L1* and *Bcl-2* was further confirmed by pyrophosphate sequencing method with methyltransferase inhibitor, 5-aza. The inhibitor 5-aza effectively attenuate the hypermethylation levels of *CREB3L1* and *Bcl-2* in the 30th passage of SiNPs-treated BEAS-2B cells (Fig 5).

### Genes expression of *CREB3L1* and *Bcl-2* triggered by SiNPs

**Fig 6:**
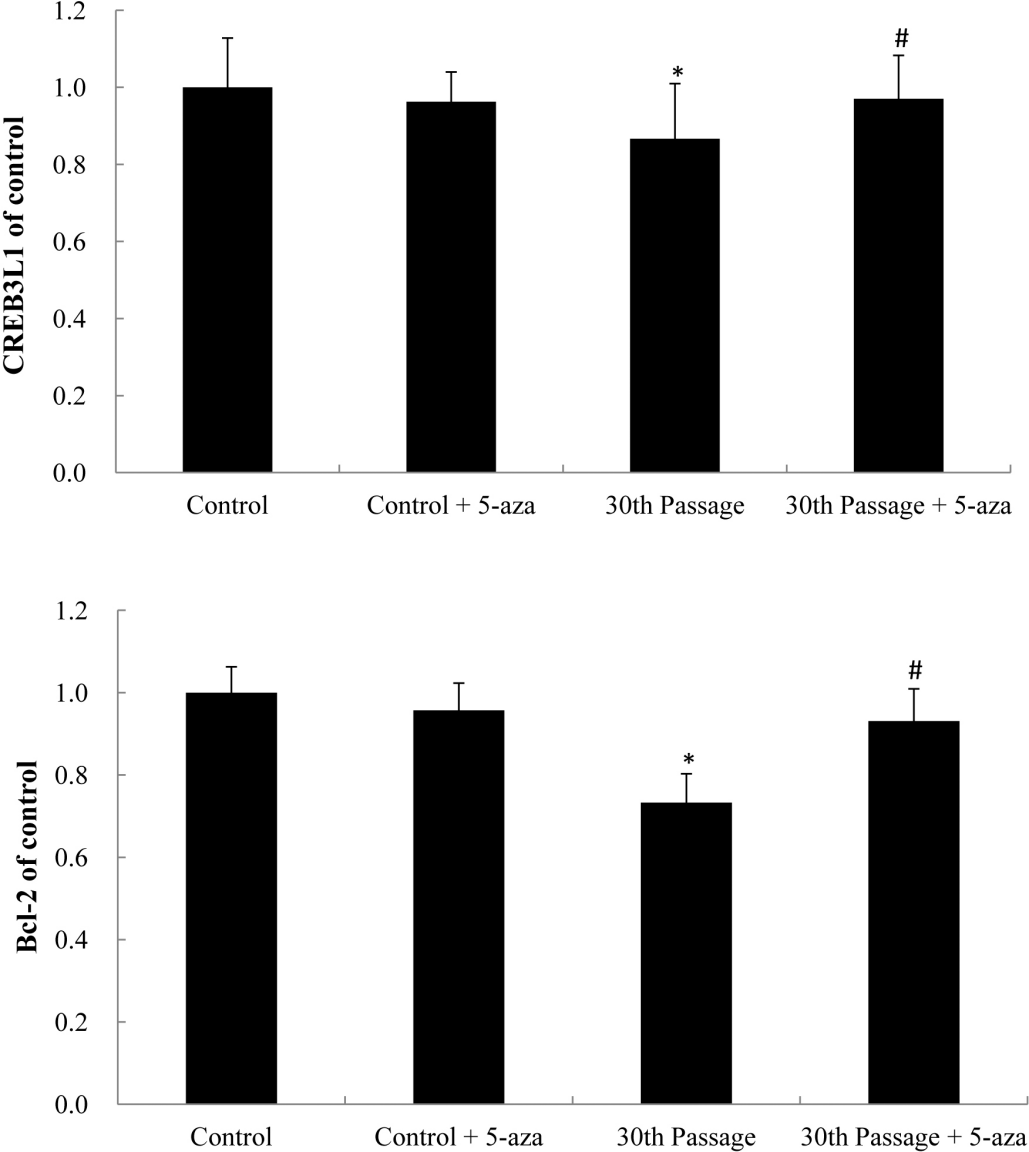
mRNA expression of *CREB3L1* and *Bcl-2* in the 30th passage of BEAS-2B cells exposed to SiNPs and the methyltransferase inhibitor 5-aza. Data are expressed as means ± S.D. *p<0.05 compared with control group. ^#^p<0.05 compared with the 30th passage of the SiNP-treated group.

To confirm whether the DNA hypermethylation regulated the expression of their relevant genes, the mRNA levels of the *CREB3L1* and *Bcl-2* were quantified by qRT-PCR analysis. As shown in Fig 5, the gene expression of *CREB3L1* and *Bcl-2* were significant downregulated in the 30th passage of SiNPs-treated groups compared to that of control, while the inhibitor 5-aza could markedly increase the mRNA levels of the *CREB3L1* and *Bcl-2* (Fig 6). Our results demonstrated that the DNA hypermethylation status of *CREB3L1* and *Bcl-2* decreased their mRNA levels of SiNPs-treated BEAS-2B cells.

### Effect of SiNPs on PI3K/Akt/CREB/Bcl-2 signaling pathway

**Fig 7:**
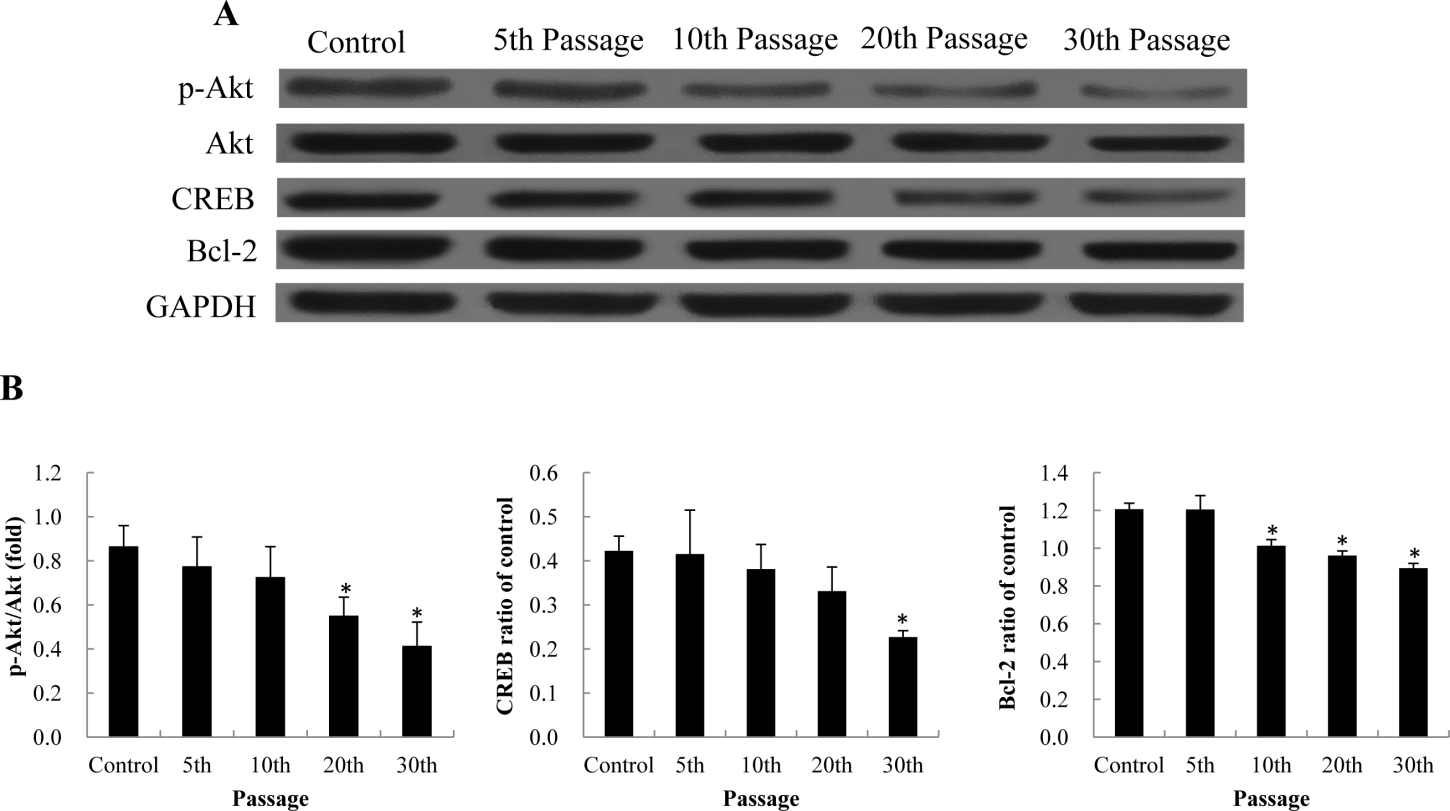
Effect of SiNPs on the PI3K/Akt/CREB/Bcl-2 signaling pathway. (A)Protein expression of p-Akt, Akt, CREB, Bcl-2. (B) Relative densitometric analysis. Data are expressed as means ±S.D.*p<0.05 compared with control group.

**Fig 8:**
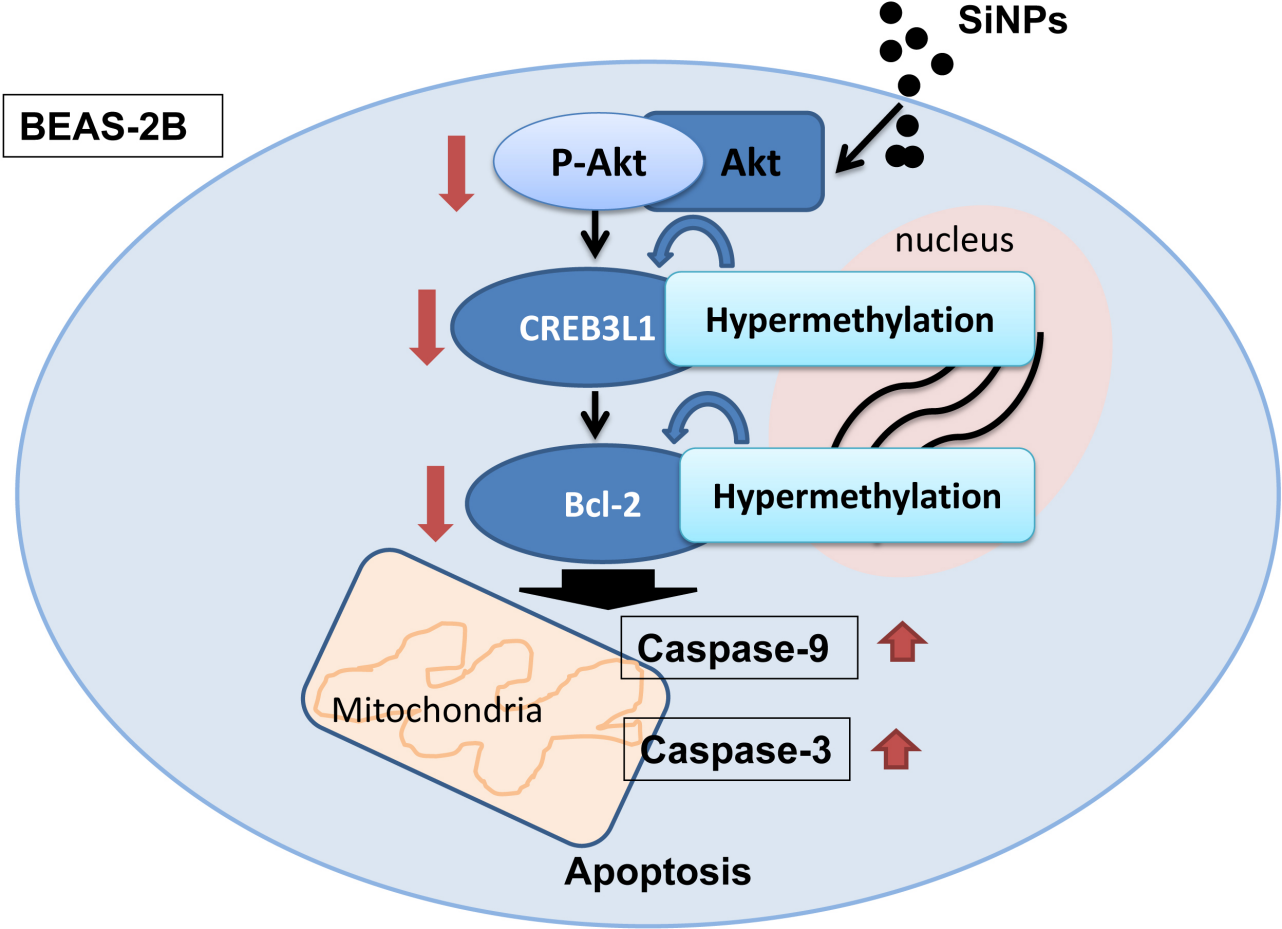
Schematic representation of the DNA hypermethylation of CREB3L1 and Bcl-2, associated with mitochondrial-mediated apoptosis via PI3K/Akt signaling pathway induced by low-dose SiNPs.

To further illustrate the underlying mechanisms of SiNPs on apoptosis, we examined the PI3K/Akt/CREB/Bcl-2 signaling pathway by western blot. As shown in Fig 7, the protein levels of p-Akt/Akt, CREB and Bcl-2 were significantly decreased in BEAS-2B cells treated with 5 μg/mL SiNPs compared to control group at all passages. Our data indicated that SiNPs triggered apoptosis via PI3K/Akt/CREB/Bcl-2 signaling pathway in a passage-dependent manner. A schematic model of the molecular mechanisms of long-term low-dose exposure of SiNPs-induced apoptosis in BEAS-2B cells is presented in Fig 8.

## Discussion

Over the last decade, nanomaterials have been shown to have the capacity to cause cytotoxicity and genotoxicity in vitro and in vivo [24–26]. Yet, there are only few studies focusing on the epigenetic toxicity mechanisms induced by nanomaterials. Many aspects of this area remain to be established, and preliminary conclusions appear contradictory [12]. DNA methylation is one of the common epigenetic mechanism resulting in changes to gene expression without DNA sequence alteration [27]. In the present study, we show for the first time that low-dose exposure of SiNPs induced apoptosis in BEAS-2B cells over 30 passages via epigenetic regulation.

Our data showed that the cytotoxicity induced by SiNPs increased in a dose- and passage-dependent manner (Fig 1). Since the cell viability is directly linked to cell death, apoptosis was evaluated by Annexin V/PI double staining. In line with the cytotoxicity results, the apoptotic rate induced by SiNPs was significantly increased in a passage-dependent manner (Fig 2). Apoptosis, is a tightly regulated form of programmed cell death, involved in an energy-dependent sequence of biochemical events that leads to cytoplasmic organelles and nuclear fragmentation as well as chromatin condensation [28]. Apoptosis occurs through both extrinsic (Fas) and the intrinsic (mitochondrial) pathways [29]. The mitochondria-mediated pathway involves a cascade of events, including the efflux of cytochrome C, the formation of apoptosomes accompanied with Apaf-1 and caspase-9, resulting in caspase-3 activation and subsequent induction of apoptosis [30]. Mitochondria-mediated apoptosis had been observed in response to several metal nanoparticles, such as copper oxide nanoparticles, silver nanoparticles and zinc oxide nanoparticles [31–33]. Here we found that the SiNPs, similarly to the nonmetal nanoparticles, could trigger the mitochondria-mediated apoptosis via the up-regulation of caspase-9 and caspase-3 in BEAS-2B cells (Fig 3). Thus, we suggested that the mitochondria-mediated apoptosis might be a common mechanism induced by nanoparticles of different types.

We adopted the HumanMethylation450 BeadChip to further analyze genome-wide methylation profiles and signaling pathways. The PI3K/Akt pathway was identified as the apoptosis-related signaling pathways among the 25 significantly altered pathways (Fig 4). The differentially methylated sites of the PI3K/Akt signaling pathway involved 32 different gene promoters, in which *CREB3L1* and *Bcl-2* were significantly hypermethylated (S3 Table). The process of DNA methylation transiently adds a methyl group to the 5’carbon position of cytosine of cytosine-guanosinedinucleotides (CpG). CpG islands are in, or near, the gene’s promoter region that allow the transcription of genes when unmethylated [34]. Our data showed that the *CREB3L1* and *Bcl-2* were significantly hypermethylated, and that the methyltransferase inhibitor 5-aza could effectively attenuate the hypermethylation levels of *CREB3L1* and *Bcl-2* (Figs 5 and 6). Tucci et, al. found that titanium dioxide nanoparticles could disturb the methionine cycle and diminish levels of methionine, indicating that titanium dioxide nanoparticles can cause DNA hypomethylation in human keratinocytes HaCaT cells [35]. Using the same cell HaCaT line, Zhuang et al. reported that silicon dioxide nanoparticles triggered global DNA hypomethylation due to the downregulation of DNMTs and MBD2 [36]. However, our results showed that the SiNPs induced more DNA hypermethylation than hypomethylation in BEAS-2B cells. We speculated that the methylation profile induced by nanoparticles can vary according to the cell line under investigation; a subject that will be important to address in future studies.

To gain insight into the underlying mechanisms of SiNPs-induced mitochondria-mediated apoptosis, we examined the PI3K/Akt/CREB/Bcl-2 signaling pathway. Our data indicated that SiNPs triggered apoptosis via PI3K/Akt/CREB/Bcl-2 signaling pathway in a passage-dependent manner (Fig 7). The surface of SiNPs contains a lot of hydroxyl radical (·OH), which has a great tendency to induce the ROS generation and oxidative damage in cells [37]. Our previous study confirmed that the ROS scavenger, N-acetylcysteine (NAC), could effectively inhibition PI3K/Akt /GSK-3β pathway induced by silica nanoparticles in L-02 cells [38]. So, we could conclude that the hydroxyl radical is a major chemical reason for the activation of PI3K/Akt pathway by SiNPs. The classic signaling pathway of PI3K/Akt regulates several pro-survival proteins, such as NF-kB, CREB and Bcl-2; as well as regulating several pro-apoptotic proteins [39]. CREB has a pro-survival effect through mediation of several transcription factors. It can directly regulate the downstream pro-survival transcription factor, Bcl-2 [40]. The Bcl-2 family is known to participate in the regulation of the apoptosis process. Bcl-2, is mainly localized in mitochondria, where it plays an important role in controlling mitochondrial membrane integrity and cytochrome C release [41–43]. Our previous study found that the PI3K/Akt signaling pathway was involved in the cross-talk between autophagy and angiogenesis [44]. In this study, the DNA hypermethylation of *CREB3L1* and *Bcl-2* induced by SiNPs was also shown to be associated with mitochondrial-mediated apoptosis via the PI3K/Akt signaling pathway. A schematic model of the molecular mechanisms obtained in this study is presented in Fig 8.

## Conclusions

The present study demonstrated that low-dose exposure of SiNPs induced cytotoxicity and mitochondrial-mediated apoptosis in a passage-dependent manner in BEAS-2B cells. The differentially methylated sites of PI3K/Akt pathway involved 32 different gene promoters, in which the *CREB3L1* and *Bcl-2* were significant hypermethylated. The DNA hypermethylation status of *CREB3L1* and *Bcl-2* were associated with a decrease in their mRNA levels. In addition, mitochondrial-mediated apoptosis was triggered by SiNPs via the downregulation of the PI3K/Akt/CREB/Bcl-2 signaling pathway. Our findings suggest that exposure to low-dose SiNPs for long-term could lead to epigenetic alteration in human bronchial epithelial cells. Our data provide persuasive evidence for further safety evaluation of nanomaterials.

## Supporting Information

S1 Table Primers for the Pyrosequencing (DNA methylation). (PDF)

S2 Table Primers for reverse transcription polymerase chain reaction analysis. (PDF)

S3 Table The primitive analysis of the gene DNA methylation of *Bcl-2* and *CREB3L1* in the 30th passage of BEAS-2B cells exposed to SiNPs. (PDF)
