# Supplementary material for: DNA Hypermethylation of CREB3L1 and Bcl-2 Associated with the Mitochondrial-Mediated Apoptosis via PI3K/Akt Pathway in Human BEAS-2B Cells Exposure to Silica Nanoparticles
Source: PLoS One. 2016 Jun 30;11(6):e0158475. doi: 10.1371/journal.pone.0158475 (PMC4928798; doi:10.1371/journal.pone.0158475)
Supplement: S1 Table — (PDF) [file pone.0158475.s001.pdf]

S1 Table. Primers for the Pyrosequencing (DNA methylation)

	**Primer Name**	**Sequence (5′ to 3')**
CREB3L1	MF	TGGAAATAGTGGAGGTGAGTATATGATG
	MR	CCTAAACCCCCAAACTAAAACAATTACC
	MS	GTGAGTATATGATGTTTTGT
Bcl-2	MF	GTATGTGGGAGTGTGTGTG
	MR	ACAACAAAACAACCCTAAAATCCCACTAC
	MS	AAAAACCACATTAACCTAAATCTTC
