# Supplementary material for: DNA Hypermethylation of CREB3L1 and Bcl-2 Associated with the Mitochondrial-Mediated Apoptosis via PI3K/Akt Pathway in Human BEAS-2B Cells Exposure to Silica Nanoparticles
Source: PLoS One. 2016 Jun 30;11(6):e0158475. doi: 10.1371/journal.pone.0158475 (PMC4928798; doi:10.1371/journal.pone.0158475)
Supplement: S2 Table — (PDF) [file pone.0158475.s002.pdf]

S2 Table. Primers for the reverse transcription polymerase chain reaction analysis

**Primer Name**	**Sequence (5′ to 3')**
GAPDH-F	TGACTTCAACAGCGACACCCA
GAPDH-R	CACCCTGTTGCTGTAGCCAAA
Bcl-2-F	TCGCCCTGTGGATGACTGA
Bcl-2-R	TCCCAGCCTCCGTTATCCT
CREB3L1-F	GCCTAAAGACGGTGGAAACG
CREB3L1-R	CCAGATCCCTGTCGTGGAA
