# Supplementary material for: DNA Hypermethylation of CREB3L1 and Bcl-2 Associated with the Mitochondrial-Mediated Apoptosis via PI3K/Akt Pathway in Human BEAS-2B Cells Exposure to Silica Nanoparticles
Source: PLoS One. 2016 Jun 30;11(6):e0158475. doi: 10.1371/journal.pone.0158475 (PMC4928798; doi:10.1371/journal.pone.0158475)
Supplement: S3 Table — (PDF) [file pone.0158475.s003.pdf]

S3 Table. The primitive analysis of the gene DNA methylation of *Bcl-2*and *CREB3L1* in 30th passages of BEAS-2B cells exposed to SiNPs.

	**Bcl-2**	**CREB3L1**
Target_ID	cg26277730	cg00352031
P.Value	9.10E-05	0.007868
Beta.Difference	0.18324	0.15204
Mean_ SiNPs-BEAS-2B	0.605241	0.600748
Mean_ BEAS-2B	0.422001	0.448705
ADDRESSA_ID	17664438	58808450
ALLELEA_PROBESEQ	ATATATAAAAATATATACACACACCTACACACACACACATTATATTACCA	TAAACTAAAAACAATAAAAATAAACACATAATACCCTATAAATATATACC
INFINIUM_DESIGN_TYPE	I	II
CHR	18	11
MAPINFO	60988085	46298408
SOURCESEQ	GTGTATGAGAGTGTGTACACGCGCCTACACACACACACGTTGTGTTACCG	CGGTATACACCCACAGGGCATCATGTGCTCACCTCCACTGTTTCCAGTCT
COORDINATE_36	59139065	46254984
STRAND	R	R
RANDOM_LOCI	NA	NA
METHYL27_LOCI	NA	NA
UCSC_REFGENE_NAME	BCL2	CREB3L1
UCSC_REFGENE_ACCESSION	NM_000633	NM_052854
UCSC_REFGENE_GROUP	TSS1500	TSS1500
UCSC_CPG_ISLANDS_NAME	chr18:60986621-60988286	chr11:46299544-46300216
RELATION_TO_UCSC_CPG_ISLAND	Island	N_Shore
ENHANCER	NA	NA
DHS	NA	NA

Note: The analysis between the group of the 30th SiNPs-BEAS-2B and normal cells. $p<0.05$ and $\beta>0.14$.
